# Identifying patterns of adaptation in breast cancer patients with cancer‐related fatigue using response shift analyses at subgroup level

**DOI:** 10.1002/cam4.1219

**Published:** 2017-10-10

**Authors:** Maxime Salmon, Myriam Blanchin, Christine Rotonda, Francis Guillemin, Véronique Sébille

**Affiliations:** ^1^ University of Nantes University of Tours INSERM SPHERE U1246 Nantes France; ^2^ University of Lorraine University of Paris Descartes EA 4360 APEMAC Nancy France; ^3^ Center Pierre Janet University of Lorraine EA4630 APEMAC/EPSAM Metz France; ^4^ INSERM CIC 1433 Clinical eidemiology – Nancy University Hospital Nancy France; ^5^ Department of Biostatistics Nantes University Hospital Nantes France

**Keywords:** Breast cancer, fatigue, patient‐reported outcome measures, response‐shift, structural equation modeling

## Abstract

Fatigue is the most prevalent symptom in breast cancer. It might be perceived differently among patients over time as a consequence of the differing patients’ adaptation and psychological adjustment to their cancer experience which can be related to response shift (RS). RS analyses can provide important insights on patients’ adaptation to cancer but it is usually assumed that RS occurs in the same way in all individuals which is unrealistic. This study aimed to identify patients’ subgroups in which different RS effects on self‐reported fatigue could occur over time using a combination of methods for manifest and latent variables. The FATSEIN study comprised 466 breast cancer patients followed over a 2‐year period. Fatigue was measured with the Multidimensional Fatigue Inventory questionnaire (MFI‐20) during 10 visits. A novel combination of Mixed Models, Growth Mixture Modeling, and Structural Equation Modeling was used to assess the occurrence of RS in fatigue changes to identify subgroups displaying different RS patterns over time. An increase in fatigue was evidenced over the 8‐month follow‐up, followed by a decrease between the 8‐ and 24‐month. Four latent classes of patients were identified. Different RS patterns were detected in all latent classes between the inclusion and 8 months (last cycle of chemotherapy). No RS was evidenced between 8‐ and 24‐month. Several RS effects were evidenced in different groups of patients. Women seemed to adapt differently to their treatment and breast cancer experience possibly indicating differing needs for medical/psychological support.

## Introduction

Cancer‐related fatigue is one of the most prevalent symptom 1 in breast cancer patients. Cancer‐related fatigue is defined as a complex subjective state characterized by a reduction in physical and mental abilities affecting cancer patients, from time of diagnosis and throughout treatment, as well as survivors 2. One of the tools for measuring cancer‐related fatigue is the Multidimensional Fatigue Inventory (MFI‐20) 3. Several studies have explored fatigue change in breast cancer patients during treatments using the MFI‐20 4, 5, 6. These studies showed heterogeneous results with different patterns of increase, decrease, or stability in fatigue over time. Moreover, all these studies assumed so‐called longitudinal measurement invariance 7 assuming that patients respond consistently on patient‐reported outcomes (PRO) and that they are directly comparable over time, which can be questioned. Indeed, in breast cancer, it is likely that patients might regularly adapt to their illness and, as a consequence, might give different answers to the questionnaires over time, not only because their fatigue has changed, but also because their perception of what fatigue means to them has changed. This phenomenon is often referred to as response shift (RS) 8 which has been hypothesized to have three different manifestations: recalibration (change in the patient's internal standards of measurements), reprioritization (change in the patient's values), and reconceptualization (change in the patient's definition of the measured concept).

In case of RS, it might be problematic or even impossible to distinguish, without appropriate methodology, change in fatigue from RS effects. The assessment of therapeutic interventions can then lead to inappropriate results, poor power to detect intervention effects and erroneous conclusions. At the same time, one can highlight the therapeutic importance of RS itself, allowing a better understanding of how patients adjust to their illness. Indeed, RS could be one of the goals of therapy in helping patients to cope with their disease and to live with it. Therefore, RS can be simultaneously viewed as measurement bias as well as an indication of a possible therapeutic benefit coming from some form of psychological adaptation or adjustment. Thus, it is important to assess the change experienced by patients by taking into account RS if appropriate, but also to detect this phenomenon and quantify it in a reliable and unbiased manner.

Most approaches proposed for RS detection and adjustment in the appraisal of change in PRO over time are performed at the sample‐level such as Structural Equation Modeling (SEM) 9 or Item Response Theory (IRT) 10. Hence, RS is considered on the overall sample of patients regardless of individual characteristics. It is thus assumed that the majority of the sample has been affected by the same change in the perception of fatigue over time. Nevertheless, we can suspect that among a sample, only some individuals might be affected by RS and different types of RS might affect different individuals to different extent. An alternative approach has been proposed 11 allowing the detection of RS and its possible time of occurrence at a subgroup level, however, it does not allow for the identification of the form of RS that might have occurred (recalibration, reprioritization, reconceptualization). For this purpose, this method could be complemented with other approaches such as SEM using Oort's procedure 9 for identifying the different forms of RS. However, to the best of our knowledge, it has never been done despite its potential for detailed RS identification at a subgroup level.

Our objective was to identify the possible different patterns of RS in a sample of breast cancer patients. A combination of methods was proposed to assess the occurrence of RS in the evolution of self‐reported fatigue over time and to identify subgroups of patients regarding different patterns of two RS processes (recalibration, reprioritization) to disentangle the contributions of RS effects and latent fatigue (unobserved fatigue level of patients) changes to the observed change.

## Method

### Data collection

The FATSEIN study 12 included breast cancer patients recruited in the cancer care centers of Nancy, Dijon, and Strasbourg in France and followed up for 24 months after surgery. Informed consent was obtained from all individuals participants included in the study. Eligibility criteria included women newly diagnosed with invasive breast cancer, undergoing breast surgery as primary treatment, age ≥18 years, without history of other cancer, no other major disabling medical or psychiatric conditions, no previous chemotherapy or radiotherapy, no metastases, and no inflammatory breast cancer. The study has been approved by the institutional review board and is registered at www.clinicaltrials.gov (NTC01064427).

Patients were recruited for a 2 year‐follow‐up including 10 visits (Appendix 1). Socio‐demographic and medical data were collected before surgery. Cancer‐related fatigue was measured with the MFI‐20 13 over the 10 visits. This questionnaire explores four domains of fatigue (physical or mental fatigue, reduction in activities or motivation) and provides a global fatigue score. Higher scores indicate more reported fatigue. Patients also completed the QLQ‐C30 quality of life (QoL) questionnaire 14 and the State‐Trait Anxiety Inventory (STAI‐State) to measure transient anxiety 15 over time. The STAI‐Trait 15 and the LOT (LOT) 16 were completed at inclusion to measure enduring levels of trait anxiety and optimism. Higher scores for the State‐Trait Anxiety Inventory and for the LOT indicate more reported anxiety and optimism, respectively.

### Statistical analysis

**Figure 1:**
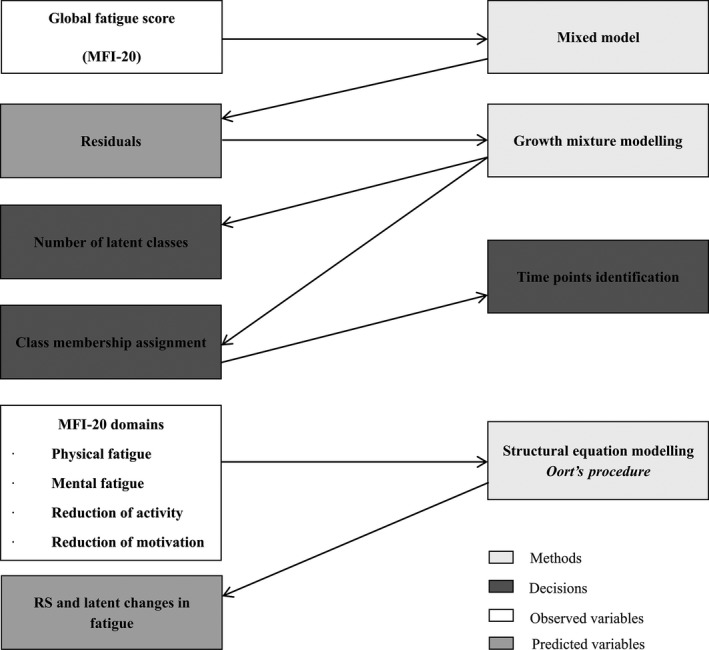
Sequential steps for response shift (RS) analyses.

Several steps were performed (Fig. 1). SAS version 9.4 and Mplus version 7.2. were used. Two‐sided *P* values<0.05 were considered statistically significant.

Firstly, the change in global fatigue score of the MFI‐20 was assessed using a mixed model from which the residuals were retained. The final model was selected using an ascending strategy where time effect and sociodemographic covariates were added according to Wald tests (*P* < 0.05). Since it was expected that chemotherapy treatment could modify fatigue change, it was added as a fixed effect, as well as its interaction with time. The addition of random effects on the intercept or the slope was assessed using a likelihood ratio test (LRT). The studentized residuals were obtained by dividing the conditional residuals of the mixed model by the estimate of their standard deviation; they were subsequently centered by removing their mean for each subject. This allowed pulling out the effect of a poor fit (high or low residuals). Following Mayo et al. 11., the change in residuals quantifying the difference between the observed global fatigue and what would have been predicted based on all the variables significantly associated with fatigue change was considered as an indicator of RS. It was assumed that the fluctuation in a patient's residuals over time might be due to RS. Hence, we considered that a subject with centered residuals close to zero for all measurement occasions did not experience RS.

Secondly, different latent classes (LC) of homogeneous centered residuals growth trajectories were identified as well as the time points where RS was expected to occur using Growth Mixture Models (GMM) 17. A categorical latent variable representing the different classes was included in the model and LC membership was inferred from data. The growth parameters (intercept and slope) could be different in each LC. The selection of the best fitting GMM and of the number of classes relied on the sample size–adjusted Bayesian information criteria (SABIC) 18. We constrained the minimum sample size to be at least 10% of the total sample size in all LC to enhance SEM convergence while keeping a maximum of different possible clinically meaningful trajectories. When the number of LC was identified, posterior probabilities of LC membership were estimated for all patients. Each patient was assigned to the LC with the highest posterior probability 17. Lastly, the time points between which RS could be experienced by patients were determined using the highest range of the residuals in each LC, and the treatment history that could have triggered RS. The distribution of the socio‐demographic and medical variables, QoL, optimism and anxiety scores measured at inclusion and over time were compared between the LC using analysis of variance (ANOVA), chi‐square tests, and mixed models.

Thirdly, the occurrence of reprioritization, uniform/non‐uniform recalibration RS in each of these LC and its effect on observed fatigue changes measured by the scores of the four domains of the MFI‐20 between the previously selected time points were assessed using SEM 9 following a four‐step method which is briefly described. This procedure was performed on the overall sample and in each LC allowing estimating three components of changes: observed changes, RS and latent change contributions to the observed change. Mathematical formulation of the SEM model, models’ identification constraints, and operationalization of RS (RS parameters) appear in Appendix 2.

#### Step 1

**Figure 2:**
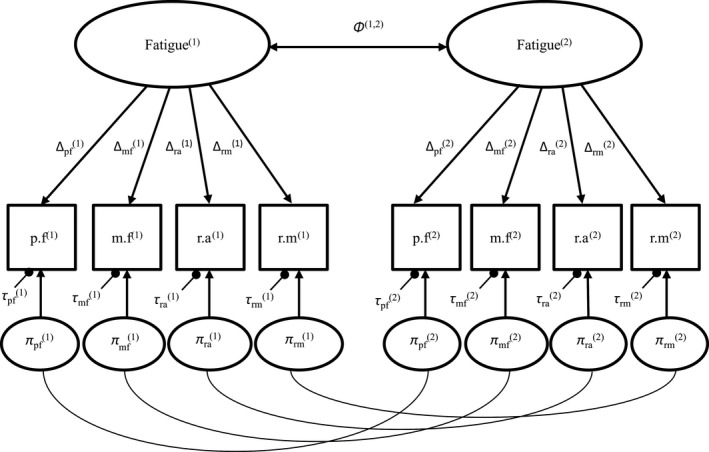
Measurement model in step 1. $\pi_{d}^{\left(t\right)}$, $\tau_{d}^{\left(t\right)}$ $\Delta_{d}^{\left(t\right)}$: unobserved residuals variances, intercepts, factor loadings of domain d at time t, respectively. Φ^(1,2)^: across occasion covariance of the latent construct of fatigue.

A “measurement model” (model 1) is first estimated (Fig. 2) with no across measurement constraints for RS parameters: factor loadings (reprioritization RS), intercepts (uniform recalibration RS) or residual variances (non‐uniform recalibration RS). The common factor is measured at two measurement occasions (Fatigue^(1)^, Fatigue^(2)^); it is assumed to explain the relationships between the observed variables (domain scores at time t: physical fatigue (p.f^(t)^), mental fatigue (m.f^(t)^), reduction in activities (r.a^(t)^), reduction in motivation (r.m^(t)^)). The model fit was considered acceptable if root mean square error of approximation index (RMSEA) was <0.08, comparative fit index (CFI) > 0.95, Tucker‐Lewis Index (TLI) > 0.95 and standardized root mean square residual index (SRMR) ≤0.08 19; across time correlations between domain scores were possibly added to reach satisfactory fit.

#### Step 2

A “no RS model” (model 2) is estimated with across measurement constraints for all RS parameters. An overall test for RS is performed with a LRT (model 1 vs. model 2). If the LRT is significant, RS is assumed. In that case, the types of RS are identified and estimated in step 3. Otherwise, overall RS has not been detected. In this case, the most parsimonious model 2 is kept, step 3 is skipped to go to step 4.

#### Step 3

Starting with model 2, step‐by‐step inspection of RS parameters that significantly improve model fit gives model 3. Across time correlations between residual factors are possibly added followed by residual factor correlations at the same measurement occasion, until satisfactory fit is reached.

#### Step 4

This final step allows estimating and testing the latent change in fatigue (model 4). The effect sizes 9 of the latent change and of RS are computed as shown in Appendix 2. Of note, non‐uniform recalibration is related to a change in residual variances that does not affect the mean observed change; it may indicate that the change in the scaling of the responses is not in the same direction or that it differs between patients.

## Results

### Sample characteristics

A sample of 466 women was recruited. Their mean age was 57 years, they were usually married (75 %) with 1 or 2 children (63 %) and living with their families (80 %). Women were mostly affected by a stage I and II cancer (84%). Most of them had a lumpectomy (78 %), a sentinel node surgery (50 %), and a neo‐adjuvant treatment based on radiotherapy with or without chemotherapy and hormone therapy.

### Change in global fatigue, in residuals, and identification of the latent classes

The variables significantly associated with fatigue change were time, chemotherapy and marital status. Global fatigue significantly changed over time (*P* < 0.001). It increased from baseline to 4‐month, remained stable between 4‐ and 8‐month and decreased during the last months of follow‐up. At 4‐ and 8‐months follow‐up, married women treated with chemotherapy reported more fatigue than married women without chemotherapy on average. At the last follow‐up, the mean global fatigue scores were close whatever the treatment group.

**Figure 3:**
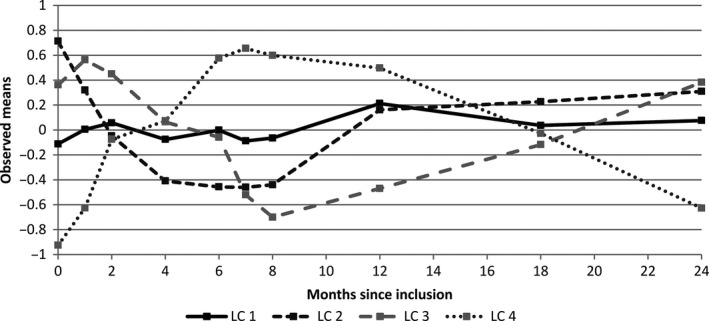
Evolution over time of the observed means of the centred residuals in each latent class.

**Figure 4:**
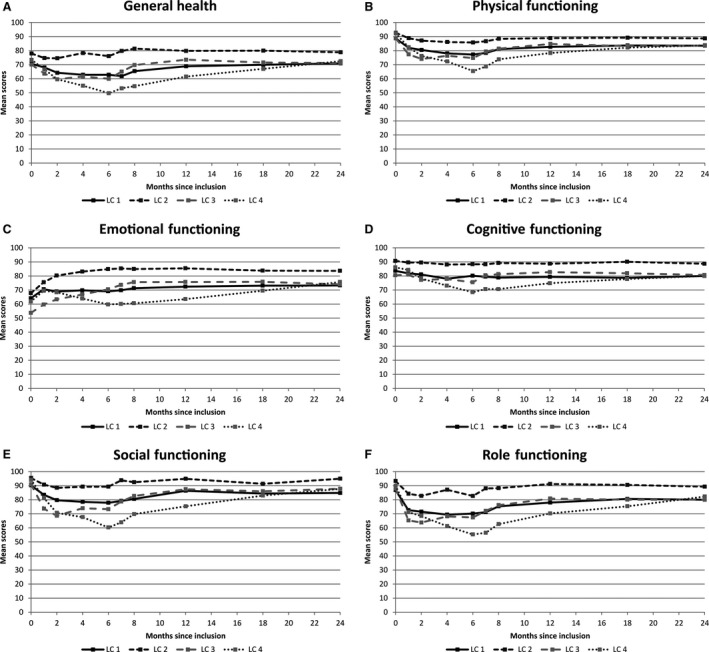
Change in the scores of the functional domains of the QLQ‐C30 quality of life questionnaire over the follow‐up.

A model with four LC (LC1‐LC4) displayed the lowest SABIC and satisfied the required minimum sample size in each LC. Figure 3 shows the estimated means of the centered residuals at each time of measurement for the four LC. There were 154 (33%) women in LC1, 61 (13%) in LC2, 118 (25%) in LC3, and 112 (24%) in LC4. Twenty‐one patients could not be assigned to a LC because of missing data on marital status (*n* = 9), treatment group (*n* = 9) or both (*n* = 3). At inclusion (Table 1), the mean ages were significantly different (*P* < 0.05) between LC1 (58.3 years) and LC4 (54.4 years), and there were more patients without chemotherapy in LC1 (*n* = 83, 54%) as compared to other LC especially LC4 (*n* = 42, 38%). The mean enduring levels of anxiety measured by the STAI‐Trait were not significantly different between the LC (*P* = 0.33) but the optimism scores of the LOT were lower in LC3 as compared to LC2 (*P* < 0.05). During follow‐up, change in the observed transient anxiety levels measured by the STAI‐State was not significantly different between the LC (*P* = 0.16). In contrast, the observed changes in all the functional domains of the QoL questionnaire QLQ‐C30 were significantly different over time between the LC (Fig. 4). The observed mean scores in LC2 were usually higher compared to the other LC (especially LC4) and quite stable over time for the following dimensions: General Health (GH, Fig. 4A), Physical Functioning (PH, Fig. 4B), Cognitive Functioning (CF, Fig. 4D), Social Functioning (SF, Fig. 4E), Role Functioning (RF, Fig. 4F). Conversely, for these dimensions, in LC4, the observed mean scores fluctuated more and usually decreased from baseline to 8‐month and subsequently increased until the end of follow‐up either reaching their initial baseline level (for the GH dimension) or remaining below it (on average −8 points for SF, −7 for CF, −10 for PF and RF at 24‐month as compared to baseline). Moreover, the difference between the observed mean scores in LC4 and LC2 (LC4 minus LC2) ranged from −3 points at baseline to −22 at 8‐month, and was −5 points at the end of follow‐up on average. In latent classes LC1 and LC3, the mean scores change in the GH, PH, CF, SF, and RF dimensions were usually close to one another. In contrast, for the Emotional Functioning (EF, Fig. 4C) dimension, at baseline, the observed mean scores were lower in LC3 as compared to the other LC (−14 points, −10, and −8 on average as compared to LC2, LC1, and LC4, respectively, *P* < 0.05). The mean scores subsequently increased in LC2 and LC3 until the end of follow‐up (+16 and +20 points in LC2 and LC3 on average, respectively) but remained lower in LC3 as compared to LC2 (−9 points on average) at 24‐month.

**Table 1:** Socio‐demographic and medical characteristics of study population at baseline in the whole sample and in each latent class

Characteristics	Whole sample *N* = 466	Number of missing values	LC1 (*N* = 154)	LC2 (*N* = 61)	LC3 (*N* = 118)	LC4 (*N* = 112)	*P*‐value
*Socio‐demographic*							
Age	57.0 (10.4)	9	58.3 (10.8)	58.3 (11.0)	56.9 (9.6)	54.4 (10.1)	0.018^a^
Marital status		12					
Single	34 (8%)		15 (10%)	7 (12%)	5 (4%)	6 (5%)	
Widowed	36 (8%)		15 (10%)	5 (8%)	9 (8%)	5 (5%)	0.414
Divorced	45 (10%)		17 (11%)	4 (7%)	13 (11%)	11 (10%)	
Married	339 (75%)		107 (70%)	45 (74%)	91 (77%)	90 (80%)	
Number of children		8					
None	48 (11%)		14 (9%)	6 (10%)	15 (13%)	10 (9%)	
1 or 2	287 (63 %)		92 (60%)	39 (65%)	74 (63%)	73 (66%)	0.813
>2	123 (27 %)		48 (31%)	15 (25%)	29 (25%)	28 (25%)	
Educational level		58					
End of the compulsory school	213 (52 %)		72 (52%)	30 (59%)	57 (53%)	47 (48%)	
High school degree	85 (21 %)		27 (19%)	11 (22%)	19 (18%)	27 (28%)	0.516
Higher education	110 (27 %)		40 (29%)	10 (20%)	32 (30%)	24 (25%)	
Employment status		9					
Employed	274 (60 %)		83 (54%)	34 (56%)	67 (57%)	80 (73%)	
Unemployed	40 (9 %)		18 (12%)	4 (7%)	12 (10%)	5 (5%)	0.053
Pensioner	143 (31 %)		52 (34%)	23 (38%)	39 (33%)	25 (23%)	
*Medical*							
Type of breast surgery		3					
Lumpectomy	362 (78 %)		125 (81%)	46 (75%)	89 (75%)	90 (80%)	0.596
Mastectomy	101 (22 %)		29 (19%)	15 (25%)	29 (25%)	22 (20%)	
Stage of cancer		18					
Stage I	239 (53 %)		87 (58%)	28 (47%)	55 (47%)	59 (54%)	
Stage II	180 (40 %)		54 (36%)	29 (48%)	53 (46%)	42 (38%)	0.490
Stage III	29 (7 %)		8 (5%)	3 (5%)	8 (7%)	9 (8%)	
Chemotherapy		12					
No Chemotherapy	206 (45 %)		83 (54%)	29 (48%)	48 (41%)	42 (38%)	0.037
Chemotherapy	248 (55 %)		71 (46%)	32 (53%)	70 (59%)	70 (63%)	
*PRO measures*							
Trait anxiety *STAI‐Trait*	47.9 (4.5)	40	47.7 (4.4)	47.2 (4.7)	48.3 (4.6)	48.2 (4.4)	0.333
Optimism LOT	19.8 (5.4)	77	20.4 (4.9)	21.1 (5.3)	18.7 (5.4)	19.6 (5.8)	0.035^b^

LC1: latent class 1, LC2: latent class 2, LC3: latent class 3, LC4: latent class 4. PRO: Patient‐Reported Outcomes; Mean (standard deviation) for continuous data, frequency (percentage) for categorical data;

a ANOVA, post hoc tests significant between LC1 and LC4;

b ANOVA, post hoc tests significant between LC2 and LC3; categorical data were compared with chi‐square tests.

Based on the mean trajectories of the centered residuals in the different LC (Fig. 3), and taking into account the end of the chemotherapy treatment (8‐month after surgery), baseline and 8‐month, and 8‐ and 24‐month after surgery were considered as the two time periods where RS could have occurred.

### Response shift analyses

#### Baseline to 8 months after surgery

Appendix 3 shows how the SEM fitted during the four‐step method proposed by Oort 9 (from model 1 to final model 4). In each LC, the fit of model 4 was satisfactory, all the fit indices were acceptable: RMSEA < 0.08, CFI > 0.95, TLI > 0.95, and SRMR ≤0.08. Table 2 shows the results of the RS analyses using SEM 9 in each latent class and in the whole sample. The types of RS that have been identified in the different latent classes and in the whole sample are shown as well as the effect sizes of the contributions of RS and latent changes to the observed changes (assuming that RS contribution + latent change contribution ≅ observed changes). Several forms of RS were identified in each LC and in the whole sample. In LC1, reprioritization (decrease of the factor loadings) was detected. Thus, 8 months after surgery, women considered physical fatigue as less important to characterize the latent construct of fatigue as compared to baseline. Uniform recalibration in physical fatigue and mental fatigue was also detected (decrease in the intercepts). It indicated that women tended to score lower on the items of physical or mental fatigue at 8 months compared to baseline despite an increase in the mean level of latent fatigue (effect sizes = 0.48 and 0.51 for latent changes in physical and mental fatigue, respectively). The effect sizes of the observed changes were thus both reduced by RS effects (effect sizes = 0.17 and 0.12 for observed changes in physical and mental fatigue, respectively). Finally, a decrease in the residual variance of the mental fatigue at 8 months was evidenced (non‐uniform recalibration).

**Table 2:** Response shift analyses using Structural Equation Modeling in the four latent classes and the whole sample between baseline and 8 months after surgery

Latent class	Response shift			Effect size		
	R	UR	NUR	Observed changes	RS contribution	Latent change contribution
LC1						
Physical fatigue	x	x		0.17	−0.32	0.48
Mental fatigue		x	x	0.12	−0.38	0.51
Activity reduction				0.44		0.44
Motivation reduction				0.42		0.42
LC2						
Physical fatigue	x			0.72	0.11	0.61
Mental fatigue		x		0.27	−0.34	0.61
Activity reduction				0.55		0.55
Motivation reduction			x	0.50		0.50
LC3						
Physical fatigue	x			0.53	−0.11	0.64
Mental fatigue		x		0.40	−0.34	0.74
Activity reduction				0.61		0.61
Motivation reduction				0.55		0.55
LC4						
Physical fatigue	x			0.36	−0.23	0.58
Mental fatigue		x		0.38	−0.26	0.63
Activity reduction				0.54		0.54
Motivation reduction			x	0.63		0.63
Whole sample						
Physical fatigue	x			0.41	−0.08	0.49
Mental fatigue		x	x	0.27	−0.25	0.52
Activity reduction		x		0.59	0.14	0.45
Motivation reduction			x	0.43		0.43

LC1, latent class1; LC2, latent class 2; LC3, latent class 3; LC4, latent class 4; R: reprioritization, UR: uniform recalibration, NUR, non‐uniform recalibration, RS: response shift.

In LC2, reprioritization was evidenced in physical fatigue (increase in the factor loadings); this domain was more indicative in the construct of latent fatigue at 8 months of follow‐up as compared to baseline. Effect sizes of observed changes were thus higher than those of latent changes. Uniform recalibration in mental fatigue was also observed (decrease in the intercepts). The observed mental fatigue effect size was thus reduced by RS. Lastly, non‐uniform recalibration was evidenced with an increase in the residual variance of the domain “Motivation reduction” at 8 months.

In LC3 and LC4, decreases in the factor loadings of the physical fatigue and in the intercepts of the mental fatigue were observed. Moreover, in LC4, non‐uniform recalibration occurred (increase in the residuals variances of the domain “Motivation reduction”). In these LC, both observed mental and physical fatigue changes were reduced by RS effects. Thus, in all LC, observed changes in physical and mental fatigue underestimated changes in latent fatigue, except for physical fatigue in LC2.

**Figure 5:**
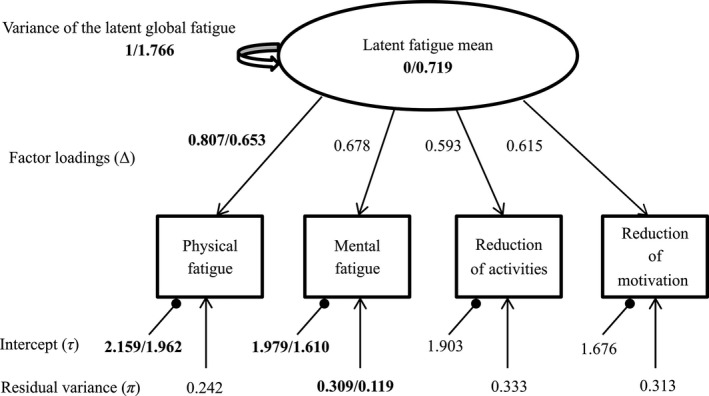
Parameters estimates of model 4 in latent class 1 between baseline and 8 months after surgery.

Furthermore, in LC1 and LC4, at 8‐month of follow‐up, reprioritization RS effects lead all factor loadings to become closer to each other meaning that women gave the same importance to all domains of fatigue at follow‐up. The parameters estimates of model 4 for LC1 are shown in Figure 5. Parameters separated by a slash represent first and second measurement occasion estimates; all other parameters were equal across measurement occasions. Three types of RS were detected indicated in bold: reprioritization in physical fatigue (decrease in factor loadings), uniform recalibration in mental and physical fatigue (decrease in the intercepts) and non‐uniform recalibration in mental fatigue (decrease in the residual variances).

In summary, the changes in the mean level of latent fatigue in all dimensions increased in all LC (all the effect sizes were positive). However, it seems that LC1 corresponded to the patients with the lowest increase in fatigue (effect sizes ranging from 0.42 for “reduction in motivation” to 0.51 for “mental fatigue”) as compared to the other LC (effect sizes ranging from 0.50 for “reduction in motivation” in LC2 to 0.74 for “mental fatigue” in LC3). Moreover, it also seems that the contribution of RS was more important (in absolute value) in LC1 as compared to the other LC for “physical fatigue” (−0.32 in LC1 and ranging from −0.11 to +0.11 in the other LC) and “mental fatigue” (−0.38 in LC1 and ranging from −0.34 to −0.26 in the other LC). The only positive effect size of RS contribution was observed in LC2 for “physical fatigue” which became more indicative of the fatigue construct (reprioritization) but it was counterbalanced by uniform recalibration in “mental fatigue” which reduced the observed change in this dimension (0.27) which was lower than the observed changes in LC3 (0.40) and LC4 (0.38). Finally, uniform recalibration in “mental fatigue” was the lowest in LC4 in absolute value (RS contribution = −0.26) as compared to the other LC (RS contribution ranging from −0.34 to −0.38).

Lastly, in the whole sample, reprioritization was detected for physical fatigue (decrease in the factor loadings), uniform recalibration for mental fatigue (decrease in the intercepts) and reduction in activities (increase in the intercepts). Non‐uniform recalibration was also detected for mental fatigue and reduction in motivation with a decrease and an increase in the residual variances, respectively.

## Eight months after surgery to the end of the follow‐up

The test of overall RS was not significant for all LC and the whole sample; hence RS was not evidenced between these two time points. The latent and observed changes were therefore the same. A decrease in fatigue was observed with the following effect sizes: −0.19, −0.03, −0.41, −0.58, and −0.29 in LC1, LC2, LC3, LC4, and in the whole sample, respectively.

## Discussion

Different patterns of RS effects on self‐reported cancer‐related fatigue were identified in subgroups of breast cancer patients. Four LC were identified where different forms of RS and effect sizes were evidenced between baseline and 8 months after surgery (1 month after the last cycle of chemotherapy). In contrast, no RS was evidenced between 8 and 24 months after surgery. In all LC and in the whole sample an increase in the latent fatigue was evidenced over the 8‐month follow‐up, followed by a decrease between 8‐ and 24‐month. During the first time period (from baseline to 8 months), the LC can be tentatively characterized as follows. LC1 corresponds to the oldest patients receiving less chemotherapy and showing the lowest increase in latent fatigue and the highest RS effects. In contrast, LC4 corresponds to the youngest patients receiving more chemotherapy, showing the lowest recalibration RS effects, and usually the poorest QoL between 4 and 8 months. Other studies using latent class analysis to identify subgroups of cancer patients with differing symptom experiences have also reported that, as compared to older patients, younger patients often display the highest levels of symptoms and the lowest QoL levels which could be explained by the fact that older patients might receive less chemotherapy and that they could be more prone to RS 20, 21. However, the reverse has also been reported regarding the association between RS and younger age 22 and more research is needed on this topic. LC2 was the smallest latent class and comprised patients with the same age mean as those in LC1, the highest mean optimism score (*P* < 0.05 as compared to LC3), usually the highest levels of QoL over the follow‐up and for whom reprioritization RS went in an opposite direction as compared to the other LC indicating that “physical fatigue” became more indicative of the fatigue construct at 8‐month compared to baseline but with recalibration RS in “mental fatigue” also being evidenced as in the other LC. LC3 comprised patients with the highest increase in latent fatigue (“physical,” “mental fatigue,” and “activity reduction”), the same recalibration RS effects in “mental” fatigue” as compared to LC2 but with a lower mean optimism score and a lower QoL at baseline regarding emotional functioning as compared to the other LC. During the second time period (from 8 to 24 months), a decrease in fatigue was observed in all LC but it was more marked in LC4 (effect size = −0.58) as compared to the other LC where the effects sizes ranged from −0.03 in LC2 to −0.41 in LC3. Hence, during this time period after the end of treatments, patients in LC4 who were younger seem to have a better ability to recover from fatigue.

During the first time period, in all LC and the whole sample, uniform recalibration RS was evidenced in mental fatigue. This led women to score lower 8^ ^months after surgery as compared to baseline on this domain, despite a mean increase in latent fatigue. Uniform recalibration was also detected for physical fatigue in the same direction but only in LC1. Such uniform recalibration RS could indicate some form of psychological adjustment such as adaptation 23 to the mental and physical fatigue experienced by women over time leading them to score lower on these domains after the last cycle of chemotherapy despite an increasing level of fatigue. Recalibration RS in fatigue was similarly observed in other studies in breast cancer patients where the then‐test approach 22, 24 was mostly used. This approach uses a retrospective (then‐test) self‐assessment of the pretest level initially measured at baseline along with a posttest assessment (e.g., 8 months after surgery). In this approach, the differences between pretest and then‐test assessments and between posttest and then‐test assessments are assumed to represent recalibration RS and latent change, respectively. Due to its simplicity, this approach is still used but has some pitfalls such as requiring an additional questionnaire, detecting only recalibration RS, being prone to recall bias 25, 26. Thus, SEM 9 or other approaches such as IRT 10 are sometimes now more often preferred.

In the 1st, 3rd and 4th LC, reprioritization RS was detected in the physical fatigue domain which became less indicative of the latent construct of fatigue at month 8 compared to baseline whereas it went in the opposite direction in LC2. Reprioritization RS was also observed in other studies focusing on QoL in prostate and breast cancer patients 27, 28 where change in priorities and importance given to social or physical domains were evidenced over time as being more or less indicative of QoL or subjective well‐being.

In all these studies RS was investigated at a sample level, assuming that this phenomenon is experienced in the same way for the majority of the sample which might not be very realistic. One can indeed expect that there is significant variability between patients due to different experiences and personality traits and that RS, often related to patients’ adaptation to illness might not have similar manifestations in all individuals. To date, quite a few studies examined RS at more individual level aside from Mayo et al. 11. who used mixed models and GMM in a sample of stroke patients but without distinguishing the different forms of RS and Blanchin et al. 29. who used IRT and Guttman errors 30 to detect discrepancies in respondent's answers compared to some expected response pattern (e.g. no RS) in a sample of hospitalized chronically ill patients. In the latter, RS was investigated at item‐level within a single dimension of the SF‐36 questionnaire (General Health).

In our study, different forms of RS could be assessed at a sub‐sample level on several dimensions of fatigue. Patients in LC1 seemed to be more prone to recalibration RS in physical and mental fatigue which could be associated with a better adaptation to illness and to its symptoms. This might related to the fact that these patients were older and received less chemotherapy. In contrast patients in LC4 who were younger and received more chemotherapy displayed the lowest recalibration RS and poor QoL. Some limitations and paths for future research can be outlined. The change in residuals was considered as an indicator of RS 11, but residuals could change over time for other reasons such as poor model fit. Nevertheless, the residuals of the mixed model were close to zero at each time so the model had a good fit (Appendix 4). The choice of the number of LC relied on the SABIC that has been shown to be more efficient than several other criteria such as AIC and BIC to detect the true number of LC in simulation studies 18. Yet, women were assigned to a LC according to their highest posterior probability. The uncertainty of classification was therefore not taken into account 31. There have been some recommendations in the mixture modeling literature to deal with this issue 32, 33 but their feasibility and performance are unknown to date in SEM for RS analyses. All estimation methods (ML and REML) assumed ignorable missing data 34. In case of non‐ignorable missing data, the probability of missingness depends on unobserved data (patients might be too tired to fill in the fatigue questionnaire) which can lead to biased estimates of RS and change in PRO 35. Although the underlying missing data mechanism is uncertain, in our sample, the comparison between patients who had missing data or not did not reveal any significant differences on covariates distributions.

The results of our study showed that RS was experienced in all LC and in the whole sample but with different forms and extent. Moreover, women also experienced strong tiredness and probably had to adapt to this situation which might be related to the uniform recalibration RS effects that were evidenced especially in older patients receiving less chemotherapy. Furthermore, in all LC, if RS had not been taken into account, observed changes in physical and mental fatigue would have underestimated changes in latent fatigue, except for physical fatigue in LC2. In conclusion, this study confirmed that RS can occur in different ways within a sample. The proposed approach allows taking into account several aspects of RS by distinguishing several groups of women who might have adapted differently to their treatment and illness possibly indicating differing needs for medical/psychological support. Special attention might be given to younger patients with more chemotherapy treatment who might have a poorer fatigue experience and QoL during therapy and could benefit from psychological support helping them to cope and to better adjust with their symptom experience.

## Conflict of Interest

No conflict of interest disclosures from any authors.

## Appendix 1

Flowchart of the treatments timeline. CT: chemotherapy; CT #: evaluation at the first day of the cycle of chemotherapy n°#; RT: evaluation just before the beginning of radiotherapy; End of RT: evaluation during the last week of radiotherapy.

## Appendix 2

### Mathematical formulation of the SEM model, models’ identification in the different steps, RS parameters, and effect sizes

#### Underlying model

This model is based on SEM. For the ith patient, we can write:

$$W_{i}=\tau +\Delta \xi_{i}+\pi_{i}$$

Where *W*_i_ is the vector of observed domain scores (physical and mental fatigue, reductions of activities and motivation), *τ* the matrix of intercepts, Δ the matrix of loading factors, *ξ*_i_ the vector of unobserved common factor (the latent construct fatigue), *π*_i_ the vector of unobserved residuals factors.

In SEM, models are usually written as mean and covariance patterns as follows:

Mean(W)=μ=τ+Δa

where a is the vector of common factor means.

The covariance of the fatigue domains can be written as

$$\text{Cov}\left(W^{\;(1)},W{}^{\;(2)}\right)=\sum =\Delta^{\left(1\right)}\Phi \Delta^{\left(2\right)}+\Omega$$

where Φ is the covariance matrix of the unobserved common factor ($\Phi =\text{Cov}\left(\xi^{\left(1\right)},\;\xi^{\left(2\right)}\right)$), and Ω the covariances matrix of the unobserved residual factors $(\Omega =\text{Cov}\left(\pi^{\left(1\right)},\;\pi^{\left(2\right)}\right)$ at the two measurement occasions (1) and (2).

The Δ and *τ* matrices can be written as:

$$\Delta =\left(\begin{array}{cc} \Delta^{\left(1\right)} & 0\\ 0 & \Delta^{\left(2\right)} \end{array}\right)\;\text{and}\;\tau =\left(\begin{array}{c} \tau^{\left(1\right)}\\ \tau^{\left(2\right)} \end{array}\right)$$

where *τ* ^(1)^ and *τ* ^(2)^ are the vectors of the intercepts and Δ^(1)^ and Δ^(2)^ the two vectors of the factor loadings at the two measurement occasions.

The mean and variance structures of the common factors are:

$$a=\left(\begin{array}{c} a^{\left(1\right)}\\ a^{\left(2\right)} \end{array}\right)\;\text{and}\;\Phi =\left(\begin{array}{cc} \Phi^{\left(1,1\right)} & \Phi^{\left(2,1\right)}\\ \Phi^{\left(1,2\right)} & \Phi^{\left(2,2\right)} \end{array}\right)$$

where a^(1)^ and a^(2)^ are the means of the two common factors (the means of the fatigue), Φ^(11)^ and Φ^(22)^ the two variances of the common factors construct at each measurement occasions, and Φ^(12)^ and Φ^(21)^ the covariances of the common factors (Φ^(12)^ = Φ^(21)^).

The matrix of residuals factor variances can be written as

$$\Omega =\left(\begin{array}{cc} \Omega^{\left(1,1\right)} & \Omega^{\left(2,1\right)}\\ \Omega^{\left(1,2\right)} & \Omega^{\left(2,2\right)} \end{array}\right)$$

where Ω^(11)^ and Ω^(22)^ are the residual factor variance‐covariance matrices at two measurement occasions, and Ω^(12)^ and Ω^(21)^ are the residual factor variance‐covariance matrices across occasions. Usually, no correlation is assumed between residuals at a given time but they can be added to improve model fit.

#### Identification of the models

In order to identify model 1 in step 1 the means of the common factor are set equal to zero, and the variances of the common factor to 1, at all measurement occasions. All other parameters are freely estimated across time. To identify model 2 in step 2, the mean and variance of the common factor are constrained to be equal to 0 and 1 at the first measurement occasion, respectively.

#### RS parameters

RS is operationalized in the following way where $\pi_{d}^{\left(t\right)},\;\tau_{d}^{\left(t\right)},\;\Delta_{d}^{\left(t\right)}$ are the unobserved residuals variances, intercepts, factor loadings of domain *d* at time *t*, respectively. Φ^(1,2)^ is the across occasion covariance of the latent construct of fatigue. Reprioritization corresponds to $\Delta_{d}^{\left(1\right)}\neq \Delta_{d}^{\left(2\right)}$ for domain d, because a change in factor loadings indicates that a domain of fatigue has become less or more indicative of the latent fatigue construct during follow‐up. Respondents could have also changed their interpretation of the response scale options during follow‐up showing recalibration. If this change occurs in the same direction and to the same extent for all items of a domain, a uniform recalibration is assumed and corresponds to $\tau_{d}^{\left(1\right)}\neq \tau_{d}^{\left(2\right)}$ If the change occurred in different directions or extents, non‐uniform recalibration is assumed and corresponds to $\pi_{d}^{\left(1\right)}\neq \pi_{d}^{\left(2\right)}$

#### Effect sizes

The estimated changes in observed fatigue (${\hat{\mu }}^{\left(2\right)}-{\hat{\mu }}^{\left(1\right)}$) is divided into three components.

$${\hat{\mu }}^{\left(2\right)}-{\hat{\mu }}^{\left(1\right)}=\left({\hat{\tau }}^{\left(2\right)}-{\hat{\tau }}^{\left(1\right)}\right)+\left({\hat{\Delta }}^{\left(2\right)}-{\hat{\Delta }}^{\left(1\right)}\right){\hat{a}}^{\left(2\right)}+{\hat{\Delta }}^{\left(2\right)}\times {\hat{a}}^{\left(2\right)}$$

where ${\hat{\tau }}^{\left(2\right)}-{\hat{\tau }}^{\left(1\right)}$ represents the contribution of the uniform recalibration to the observed changes in fatigue, $\left({\hat{\Delta }}^{\left(2\right)}-{\hat{\Delta }}^{\left(1\right)}\right){\hat{a}}^{\left(2\right)}$ the contribution of the reprioritization, ${\hat{\Delta }}^{\left(2\right)}\times {\hat{a}}^{\left(2\right)}$ the contribution of the latent changes with a^(2)^ representing the change in the construct of fatigue across time (since a^(1)^=0). The effects size of each component is computed by dividing it by the estimated standard deviation of the observed changes $\sqrt{{\hat{\sigma }}^{\left(1,1\right)}+{\hat{\sigma }}^{\left(2,2\right)}-2\times {\hat{\sigma }}^{\left(1,2\right)}}$ where ${\hat{\sigma }}^{\left(1,1\right)}$ and ${\hat{\sigma }}^{\left(2,2\right)}$ are the estimated variances, and ${\hat{\sigma }}^{\left(1,2\right)}$ an estimate of across time covariances of the observed change in model 4.

## Appendix 3

### Fit of the Structural Equation Models in the different latent classes and in the whole sample

**Table:**

		Fit index			
		RMSEA	CFI	TLI	SRMR
LC1	Model 1	0.04	0.99	0.99	0.04
	Model 2	0.12	0.91	0.91	0.20
	Model 3	0.04	0.99	0.99	0.06
	Model 4 = Model 3	0.04	0.99	0.99	0.06
LC2	Model 1	0.10	0.97	0.95	0.06
	Model 2	0.16	0.87	0.86	0.04
	Model 3	0.08	0.97	0.97	0.08
	Model 4	0.07	0.98	0.97	0.08
LC3	Model 1	0.09	0.98	0.96	0.05
	Model 2	0.11	0.94	0.94	0.15
	Model 3	0.12	0.91	0.91	0.20
	Model 4	0.07	0.98	0.97	0.05
LC4	Model 1	0.04	0.99	0.99	0.04
	Model 2	0.07	0.97	0.96	0.20
	Model 3	0.04	0.99	0.99	0.06
	Model 4 = Model 3	0.04	0.99	0.99	0.06
Whole sample	Model 1	0.08	0.97	0.96	0.04
	Model 2	0.12	0.91	0.91	0.19
	Model 3	0.07	0.98	0.97	0.04
	Model 4 = Model 3	0.07	0.98	0.97	0.04

LC, latent class; RMSEA, root mean square error of approximation; CFI, comparative fit index, TLI, Tucker‐Lewis index; SRMR, standardized root mean square residuals.

## Appendix 4

Figure: Mean of the centered residuals of the mixed model
